# Factor V Leiden Heterozygous: Complicated by Recurrent Venous Thromboembolism and Bilateral Pulmonary Embolism

**DOI:** 10.7759/cureus.63701

**Published:** 2024-07-02

**Authors:** Erica Han, Kaitlyn Burdsall, Oshea Escamilla

**Affiliations:** 1 Hematology, Edward Via College of Osteopathic Medicine, Spartanburg, USA; 2 Family Medicine, Edward Via College of Osteopathic Medicine, Spartanburg, USA

**Keywords:** coagulation factor v gene f5, anticoagulation, venous thromboembolism (vte), pulmonary embolism (pe), factor v leiden (fvl)

## Abstract

Factor V Leiden (FVL) is a hypercoagulable disorder that puts patients at increased risk of initial venous thromboembolism (VTE). However, those with heterozygote status are not usually susceptible to recurrent VTE. This is a case of a 35-year-old Caucasian male who presented to the emergency department with shortness of breath and chest pain. He had a past medical history of superficial thrombophlebitis and deep vein thrombosis (DVT) and was known to be FVL heterozygous. His home medications did not include anticoagulation medications at the time of presentation to the emergency department. The patient was diagnosed with bilateral pulmonary embolisms (PEs) secondary to a recurrent DVT. Initial treatment included a pulmonary thrombectomy and a lower extremity thrombectomy. Despite the patient being placed on heparin, there was a recurrence of the PE three days later, requiring a repeat pulmonary thrombectomy. This case of recurrent VTE in a heterozygous FVL patient is unusual and should lead to new considerations on the approach to lifelong anticoagulation in these patients.

## Introduction

Factor V Leiden (FVL) is an autosomal dominant disorder resulting from a single point mutation in the factor V (F5) gene, which eliminates the cleavage site, rendering it resistant to inactivation by activated protein C (aPC) [1]. Under normal circumstances, aPC reduces thrombin production through degradation of factor V [2]. The mutated factor V has a prolonged half-life and prolonged procoagulation activity, leading patients with FVL to be in a hypercoagulable state [3]. Many FVL patients are asymptomatic throughout their lifetime and will, therefore, go undiagnosed. A venous thromboembolism (VTE) event will often prompt an investigation into a possible coagulation disorder. FVL can be diagnosed through genetic testing, mutation testing, or functional aPC resistance assays [4].

The hypercoagulable state in a heterozygous FVL patient leads to a five- to seven-fold increased risk for an initial deep venous thrombosis (DVT) event compared to the general population [5]. There is no statistically significant difference in recurrence rates for a DVT in FVL patients compared to those in the general population without the F5 gene mutation [6]. Prophylactic anticoagulation is not indicated in asymptomatic FVL patients with no history of VTE events. In an acute VTE, treatment of FVL patients should be directed by standard guidelines depending on what type of venous thromboembolic event they are experiencing. Continued care of these patients should involve an individualized risk stratification to decide the duration of oral anticoagulation therapy [7]. Currently, indefinite anticoagulation for FVL patients is decided on a case-by-case basis, with stronger consideration for patients with homozygous FVL, unprovoked VTE, male sex, life-threatening pulmonary embolism (PE), strong family history of VTE, or more than one inherited thrombophilia [1].

This is a case of a 35-year-old male with heterozygous FVL and a history of DVT presenting with bilateral PE.

## Case presentation

A 35-year-old male presented to the emergency department (ED) with shortness of breath and chest pain with radiation to the upper back. Symptoms began two hours prior to arrival to ED when the patient awoke and noticed worsening of symptoms when reaching for a glass of water on his nightstand. The patient felt lightheaded and attempted to alleviate his symptoms with aspirin 325 mg. No improvement was noted, which prompted the patient to call an ambulance.

His past medical history was relevant for heterozygous FVL diagnosis in 2009, which was discovered after treatment of superficial thrombophlebitis and DVT. The patient was initially started on warfarin anticoagulation following this event but was noncompliant. Several years later, the patient presented with symptoms of thrombophlebitis, and warfarin was restarted. He was inconsistent with his follow-up appointments, causing difficulties with his international normalized ratio (INR) monitoring. After two years, the patient's warfarin was changed to rivaroxaban. However, five months prior to his presentation to the ED, the patient's rivaroxaban was discontinued by his hematologist due to the patient being asymptomatic.

Pertinent past medical history of hypertension, morbid obesity with a body mass index (BMI) of 48.2, chronic venous stasis, and significant alcohol and tobacco/nicotine use were noted. Despite tobacco use cessation in 2013, the patient continued to use vaporizers and nicotine packets. His pertinent family history included sleep apnea in both his mother and father and atrial fibrillation in his mother. Recent travel included a four-hour drive within the last few weeks.

On physical examination, shortness of breath, tachycardia, lightheadedness, diaphoresis, chest pain, and 1+ edema were noted present on arrival. The work-up included a trans-thoracic echocardiogram positive for increased right ventricular systolic pressure, moderately to severely dilated right ventricle, mild concentric left ventricle hypertrophy, and mild tricuspid regurgitation. Relevant labs included decreased platelets at 117 × 10^9^/L (normal: 150-400 × 10^9^/L) and D-dimer >20 g/L (normal: <0.5g/L). The electrocardiogram illustrated normal sinus rhythm. Chest CT with contrast revealed (lateral) bilateral PEs with evidence of right heart strain. Venous duplex ultrasound of the lower extremities was positive for acute DVT of the right external iliac and common femoral veins with superficial thrombophlebitis of the left greater saphenous vein.

**Figure 1:**
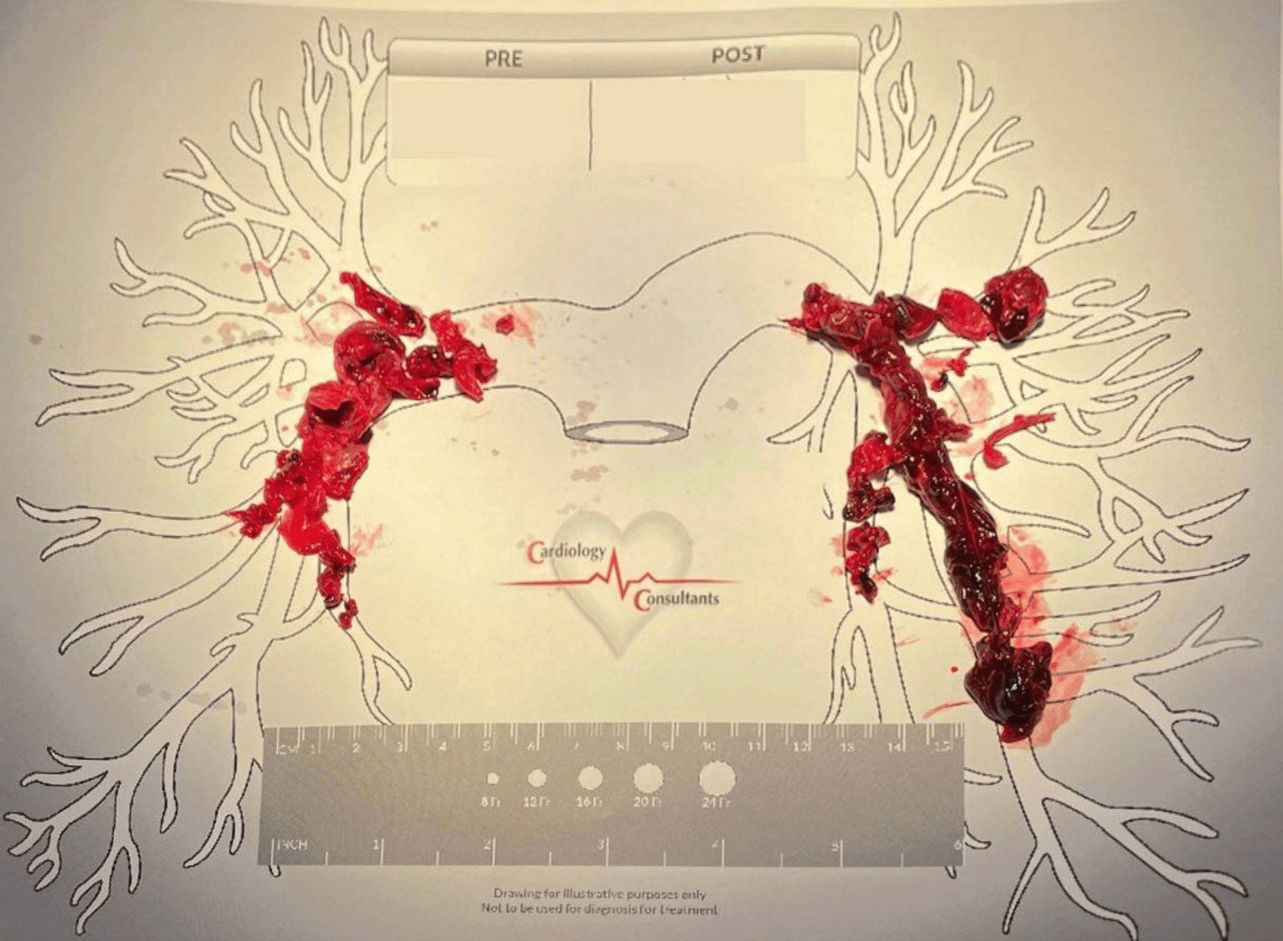
This image displays part of the patient’s total clot burden removed from two consecutive pulmonary thrombectomies performed as mentioned above. Pre-operation oxygen saturation is 90% on 6 L of oxygen. Post-operation oxygen saturation is 97% on room air.

**Figure 2:**
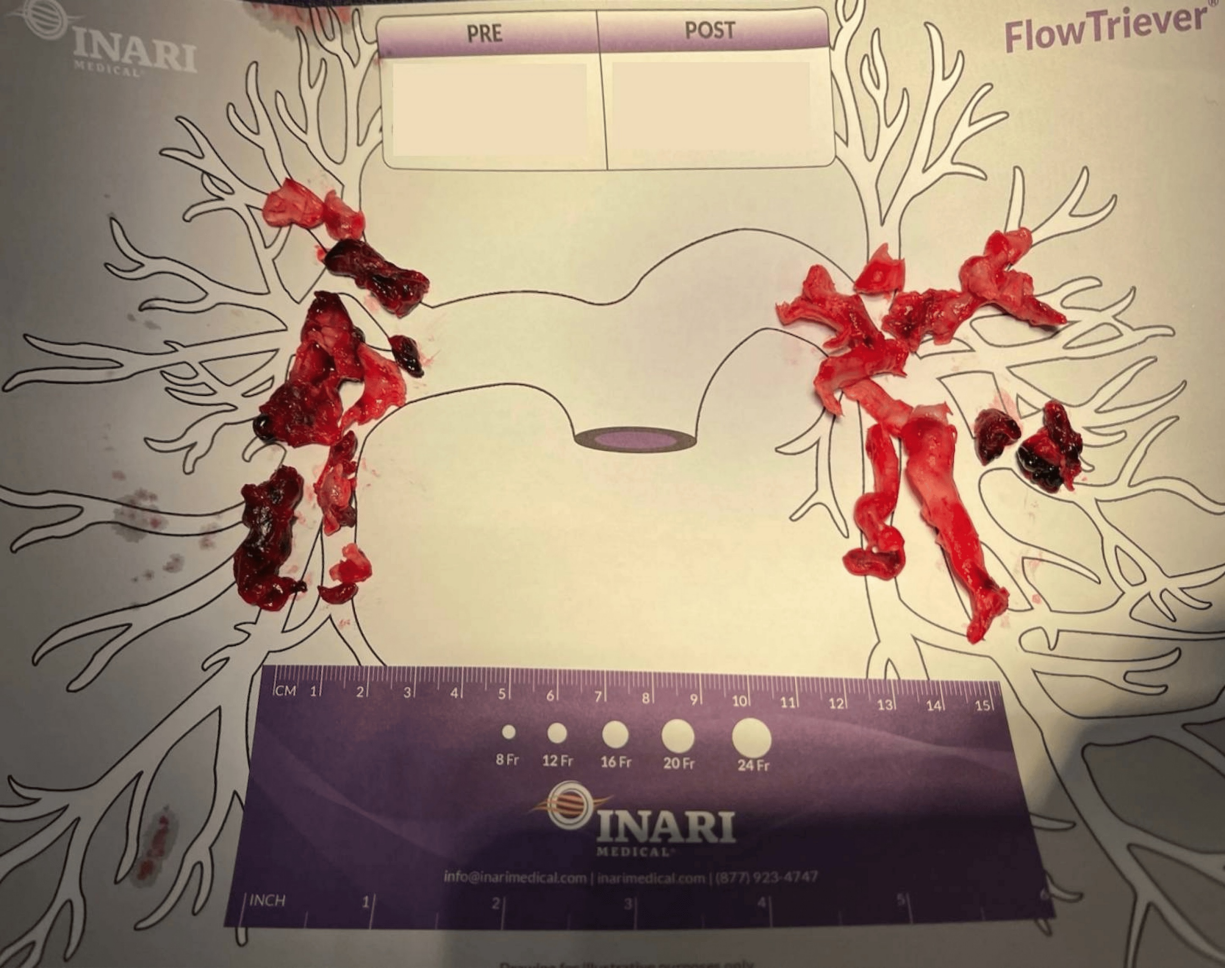
This image displays part of the patient’s total clot burden removed from two consecutive pulmonary thrombectomies performed as mentioned above. Pre-operation oxygen saturation is 89% on 10 L of oxygen. Post-operation oxygen saturation is 98% on room air.

The patient was started on a heparin drip before a bilateral pulmonary thrombectomy. The patient did not require oxygen in the ED but eventually received a high-flow nasal cannula up to 10 L before surgery due to SpO_2_ at 88%. A right lower extremity (LE) thrombectomy was performed the following day. Within one day following the LE thrombectomy, the patient displayed worsening hypoxia with an oxygen requirement of up to 13 L compared to the 6 L oxygen supplementation he received the day prior to surgery. This prompted a repeat chest CT, which showed a similar burden of bilateral PEs as his pre-surgical CT. A repeat pulmonary thrombectomy was performed, and improvement was noted with the patient's SpO_2_ at 93% on room air following the procedure. The total clot burden from the two pulmonary thrombectomy procedures is shown below in Figure 1 and Figure 2. He was discharged on apixaban 10 mg twice daily for four days, followed by 5 mg twice daily thereafter. He was instructed to follow up with his primary care provider upon discharge.

## Discussion

FVL is an autosomal dominant inherited hematologic disorder that affects thrombin degradation by protein C. This guanine to alanine single point mutation in the factor V gene is a disease found predominantly in Caucasian males of European ancestry [8]. A single copy of the FVL mutation is present in 3% to 8% of men with this background, which can cause a four to eight times increase in clot development [9]. The percentage of the population that carries two copies of the FVL mutation can be as high as one in 5,000 Caucasian men, causing an 80 times increase in clot development due to their hypercoagulable state [9].

Virchow's triad is a concept that predicts a future thrombotic event based on three overarching divisions: damage to the endothelium, stasis of blood, and hypercoagulability [10]. Many inherited, behavioral, and environmental variables can alter each category, thus increasing a patient's VTE risk. Risk factors include obesity, pregnancy, oral contraceptives, male sex, malignancy, immobilization, recent surgery in the past three months, and diabetes [11]. It is critical to consider these risk factors when performing individualized risk assessments to predict future VTE events and when managing a VTE after it has occurred. Suspicion of FVL increases in patients who have already developed a thrombus by 50 years old or those who have a family history of VTE [4].

Many patients with FVL mutations go undiagnosed as the disease does not directly cause symptoms [12]. Therefore, a diagnosis of FVL is initially guided by a comprehensive history and physical exam findings of a VTE. In these patients, testing for an FVL mutation is encouraged. An initial screening test checks the patient's blood for resistance to aPC, and a positive test indicates a 90-95% likelihood of an FVL mutation [13]. To confirm, a genetic test, also known as DNA analysis, can look for mutations of the F5 gene to help distinguish between heterozygous and homozygous status [13].

Despite a patient having a FVL diagnosis, only about 10% of these patients will develop an abnormal clot in their lifetime [9]. Therefore, lifelong anticoagulation is not currently recommended in FVL heterozygous patients who experience their first VTE event, which is in accordance with routine VTE treatment guidelines, though a case-by-case consideration may be done for those with high-risk characteristics [14]. Typically, patients with acute VTE are started on anticoagulation medications for at least six months, after which medications may be discontinued. On the other hand, patients who have had recurrent VTE events will be recommended a lifelong course of medication regardless of any other predisposing risk factors [14]. This lifelong anticoagulation can reduce the risk of recurrent VTE by up to 95% [15].

Intravenous heparin is typically administered in the hospital setting. Warfarin and factor Xa inhibitors are available as oral tablets in an outpatient setting. Although warfarin is the most cost-effective oral anticoagulant, the monitoring protocol can be challenging for some patients, which is noted by higher non-compliance rates. Warfarin can also interact with many food products and medications, as well as having a higher risk of bleeding in the patient [16]. Therefore, factor Xa inhibitors, otherwise known as direct oral anticoagulants (DOACs), are becoming more popular due to their ease of monitoring and lower risk of bleeding complications. It has been shown that there is no difference in outcome when managing FVL patients on the same types and dosages of anticoagulation medications compared to the general population; therefore, choosing the proper medication should be based on patient preference, compliance, and possible drug interactions [4].

In 2015, the patient's hematologist assessed his risk and determined a 20% chance of recurrent DVT and PE. This VTE risk stratification was guided by current literature, and all risk factors were considered. However, as mentioned above, the patient had not been on anticoagulation treatment for five months before his recent ED visit in 2023. The patient was taken off anticoagulant medication by his hematologist despite his heterozygous FVL status because it had been 14 years since his initial VTE incident, and FVL heterozygous status is thought to have no statistically significant elevation in recurrence rates for a VTE [6]. However, his heterozygous FVL diagnosis should not be overlooked when managing and preventing recurrent VTEs because of the host of other variables that contributed to his hypercoagulable state, such as hypertension, hyperlipidemia, elevated BMI, superficial thrombophlebitis, chronic right leg non-healing ulcer, recent travel, and a history of heavy alcohol and tobacco use. Physicians should not only consider the diagnosis of FVL when managing patients with a VTE but also consider the patient's overall risk of future VTE development.

Heterozygous FVL patients already have an increased risk of an initial VTE. Though data show that heterozygous FVL patients are not at an increased risk of recurrent VTE, the decision to stop anticoagulation should be based on individual risk factors. The patient presented here had several additional VTE risk factors that should have led him to remain on long-term oral anticoagulation. This VTE episode occurred only a few months after his rivaroxaban was discontinued despite his body habitus, sedentary lifestyle, and continued tobacco and alcohol use.

## Conclusions

A VTE risk assessment tool can guide physicians to classify patients into low-, moderate-, or high-risk categories by considering factors such as genetic dispositions, age, previous VTE history, recent surgery, immobilization, pregnancy, and cancer. Low-risk patients typically lack VTE risk factors, whereas those who are high-risk possess multiple risk factors simultaneously. Primary care physicians should seriously consider long-term anticoagulation in high-risk patients despite their heterozygous FVL status, especially if a hematologist is not following them. In this case, the patient had several years of inadequate anticoagulation treatment, discontinued entirely only a few months before this case, despite demonstrating numerous risk factors for a VTE event. We believe that the decision to stop his anticoagulation treatment stemmed from his status as a heterozygous mutation, which is not seen as a significant indicator of recurrent VTE. It is crucial not to disregard a heterozygous FVL mutation in the presence of outside risk factors, as this patient's case shows clear evidence of the consequences inadequate anticoagulation treatment may cause. It would be beneficial if more research was done looking into the incidence of recurrent VTE in FVL patients with comorbidities and how their treatment should differ from those who are lower risk. In this case, the 35-year-old patient suffered from not only a DVT but multiple serial PE events requiring three total thrombectomies within a span of three days. This patient had an array of risk factors for VTE as well as an FVL heterozygous status. If anticoagulation had been continued, this severe and nearly fatal VTE event that caused multiple bilateral PEs could possibly have been avoided.
